# RNA-sequencing analysis of the Diquat-degrading yeast strain *Meyerozyma guilliermondii* Wyslmt and the discovery of Diquat degrading genes

**DOI:** 10.3389/fmicb.2022.993721

**Published:** 2022-09-02

**Authors:** Fangyuan Wang, Lingwei Kong, Jing Guo, Xiuli Song, Bo Tao, Yujun Han

**Affiliations:** ^1^College of Agronomy, Northeast Agricultural University, Harbin, China; ^2^School of Geographical Sciences, Lingnan Normal University, Zhanjiang, China

**Keywords:** Diquat, *Meyerozyma guilliermondii* Wyslmt, biodegradation, RNA-Seq, qRT-PCR, prokaryotic expression

## Abstract

Diquat is used in agricultural contexts to control the growth of broadleaf and grassy weeds in both terrestrial and aquatic areas. Diquat can be readily absorbed by the soil and can remain therein for extended periods of time, altering the local microenvironment. In this study, the *Meyerozyma guilliermondii* Wyslmt yeast strain, which has the capacity to degrade Diquat, was isolated from soil exposed to long-term Diquat treatment. Over a 7-day incubation period, this strain was able to remove 42.51% of available Diquat (100 mg/L). RNA-Seq was performed to assess changes in gene expression in this yeast strain over the course of Diquat degradation, revealing 63 and 151 upregulated and downregulated genes, respectively. KEGG pathway enrichment analysis revealed these genes to be most highly enriched in the carbohydrate metabolism pathway. Through functional annotation and gene expression analyses, we identified seven genes were predicted to be involved in Diquat biodegradation. Results of qRT-PCR assays indicated that the relative mRNA expression levels of these seven genes were significantly higher relative to the control group. Together these analyses led to the identification of *DN676* as a candidate Diquat-degrading gene. When a pET-*DN676* vector was expressed in *E. coli* BL21, this strain was able to remove 12.49% of provided Diquat (100 mg/L) over the course of a 7-day incubation. These results thus confirmed that the *DN676* gene can promote Diquat degradation, with these studies having yielded an engineered BL21-pET-*DN676* bacterial strain capable of degrading Diquat.

## Introduction

Diquat (1,1′-ethylene-2,2′-bipyridylium) is a non-selective bipyridylium herbicide (Wang et al., 2017). It is widely used to impair photosynthetic activity and thereby kill exposed plants (Yuan et al., 2020). In agricultural contexts, Diquat is applied to control the growth of broadleaf and grassy weeds in both aquatic and terrestrial non-crop regions (Ducrot et al., 2010). Diquat is immediately inactivated upon reaching the soil *via* its absorption by clay minerals and organic matter. In this state, it is protected from leaching and can accumulate in the soil system. As it is highly water-soluble, establishing effective and secure procedures for purifying soil and water polluted by Diquat is crucial.

Microbial remediation is a process whereby microbes convert environmental toxicants to yield less toxic byproducts (Chen et al., 2015; Liu et al., 2015; Cycon and Piotrowska-Seget, 2016), offering a promising approach to removing chemical pollutants from contaminated soil and water biomes. Relative to traditional chemical or physiochemical approaches, microbial remediation is a more efficient means of controlling or eliminating environmental pollution (Chen et al., 2014; Arora et al., 2018; Yang et al., 2018). Both Diquat and paraquat are bipyridine herbicides, and the limited number of studies exploring their degradation to date have largely focused on paraquat. It has been established that a number of fungus species are effective paraquat degraders. For instance, *T. versicolor ECS-79*, *T. pavonia ECS-67*, and *H. dispersum ECS-705* can degrade paraquat at similar 55, 55, and 71% rates, respectively (Camachomorales et al., 2017). Funderburk (1969) found that *L. starkeyi* was able to degrade both Diquat and paraquat. Furthermore, a bacterial consortium capable of achieving 97% paraquat (100 mg/L) degradation (Li et al., 2017), while Lipomyces yeast species were able to degrade 100% of available paraquat (27 mg/L) over a 3-day culture period in liquid medium (Hata et al., 1986). These prior studies concentrated on isolating and characterizing microbes that metabolized paraquat and Diquat. However, studies regarding the metabolic genes that are involved in the degradation of Diquat are limited. In the present study, we identified the Diquat-degrading *Meyerozyma guilliermondii* Wyslmt yeast strain and found that 42.51% of provided Diquat (100 mg/L) was degraded by this strain over the course of a 7-day incubation. In order to discover the genes facilitating Diquat degradation, we further sequenced the genome of this Wyslmt strain (deposited in GenBank under the name “Wyslmt” with the accession number MZ520358; Bioproject accession: PRJNA809846). A novel degradation gene, *DN676*, was discovered to be involved in Diquat degradation, and its expression and degradation phenotype were confirmed. As such, the *DN676* gene may represent a promising candidate gene for use in future herbicide degradation efforts.

## Materials and methods

### Sample collection

Soil samples were collected from soil that had been exposed to Diquat for extended periods of time and were sieved using a 5.0 mm mesh to remove debris and loose stones. Samples were transferred into plastic bags and stored at 4°C.

### Chemicals and reagents

Diquat (99.9%) was purchased from the Putian Genesis Biotechnology co., Ltd. (Beijing, China). Other chemicals used in this study were of analytical grade, and those used for HPLC analysis were of HPLC grade. Liquid enrichment medium (EM) used for this study was composed of a 0.5 g MgSO_4_⋅7H_2_O, 1.0 g KH_2_PO_4_, and 5.0 g peptone per liter. Potato dextrose broth (PDB) medium was composed of 200.0 g potato and 20.0 g glucose per liter. Luria-Bertani (LB) broth was composed of 10.0 g peptone, 5.0 g yeast powder, and 5.0 g NaCl per liter. Diquat was added to the medium at appropriate concentrations to yield Diquat-supplemented EM, PDB, or LB (DEM, DPDB, and DLB, respectively). Medium was sterilized *via* autoclaving for 30 min at 121°C.

### Diquat-degrading microbe enrichment and isolation

To enrich for Diquat-degrading microbes, 5 g samples of soil were added to 100 mL of DEM containing 500 mg/L Diquat in a 250 mL Erlenmeyer flask, and samples were then incubated for 5 days with constant shaking (150 rpm) at 30°C under aerobic conditions. Next, 10 mL of the enriched culture was transferred into fresh DEM containing 1,000 mg/L Diquat, and this process was repeated three times to a final Diquat concentration of 2,500 mg/L. After being serially diluted, these enriched cultures were subsequently plated onto PDB agar plates with Diquat (2,500 mg/L) added and cultivated at 28°C. Then, to separate pure cultures, morphologically distinct colonies were selected and streaked onto PDB agar plates. One fungal isolate, known as strain Wyslmt, showed high activity and was thus chosen for more analysis. All strains were kept on DPDB containing 2,500 mg/L of Diquat, and they all were kept frozen in 30% glycerol stocks at −80°C.

### Characterization of the Wyslmt strain

A combination of conventional biochemical methods and 26S rDNA sequencing was used to identify the Wyslmt strain. Total genomic DNA was isolated from this fungi with a Yeast Ezup Column Yeast Genomic DNA Extraction Kit (Sangon Biotech Co., Ltd., Shanghai, China), and sequencing was performed with the NL1 (5′-GCATATCAATAAGCGGAGGAAAAG-3′) forward primer and the NL4 (5′-GGTCCGTGTTTCAAGACGG-3′) reverse primer. PCR settings were as follows: 94°C for 4 min; 30 cycles of 94°C for 45 s, 55°C for 30 s, and 72°C for 60 s; 72°C for 10 min. The resultant amplified products were separated *via* 1% agarose gel electrophoresis and purified with a SanPrep column DNAJ gel recovery kit (Sangon Biotech), after which sequencing was performed by Sangon Biotech Co., Ltd. (Shanghai, China).

After sequencing, 26S rDNA sequences were aligned and compared to those in the National Center for Biotechnology Information (NCBI) database using the BLAST program. MEGA version 6.0 was used for phylogenetic and molecular evolutionary analysis. The phylogenetic tree was built using the neighbor-joining method, and bootstrapping was done for 1,000 repetitions.

### Diquat level measurements

Dichloromethane was used three times to extract Diquat. The mixed organic extracts were subsequently concentrated, dried using an evaporator, and the volume was adjusted using methanol of the High Performance Liquid Chromatography (HPLC) grade. A 0.22 μm nylon filter was used to filter the aqueous samples after extraction in order to prepare them for HPLC analysis. The concentration of Diquat in each sample was quantified *via* HPLC (Ultimate 3000). For Diquat detection analyses, HPLC was performed using a variable wavelength UV detector set to 308 nm and fitted with a reverse-phase C18 column (4.6 × 250 mm, 5 μm) at a flow rate of 1.0 mL/min (acetonitrile/water = 40/60, v/v) and a column temperature of 30°C. All injection volumes were 10 μL.

### Calculation of Diquat degradation rate

The initial and final concentrations of Diquat were measured *via* HPLC, and the percentage of Diquat removed was calculated. The percentage of Diquat degraded was calculated as follows:


X=(C-CKC)X/C×CK100%

where X is the degradation rate of Diquat, C_*CK*_ is the original concentration of Diquat (mg/L), and C_*X*_ is the final concentration of Diquat (mg/L).

### Biodegradation assays

Wyslmt strain yeast were cultured to the logarithmic phase of growth (OD_600_ = 0.6) to prepare a stock solution from which a 1% inoculum was added into 100 mL of DPDB containing Diquat (100 mg/L). The concentration of Diquat and yeast growth were measured on days 1, 3, 5, and 7 of culture at 28°C with constant agitation (180 rpm). Yeast growth was monitored by measuring the absorbance of culture supernatants at 600 nm using a TU-1901 spectrophotometer (Beijing Purkinje General Instrument Co., Ltd., China). The concentration of Diquat was measured *via* HPLC. Experiments were repeated in triplicate, and control solutions were prepared without any starting inoculum.

### RNA-seq

For RNA-Seq analyses, a 1% Wyslmt yeast inoculum was added to 100 mL of DPDB (2,500 mg/L), with a sample without any Diquat being prepared as a control. Both treatments were prepared in triplicate, and were incubated for 24 h at 28°C with constant shaking (180 rpm) (Zhang et al., 2019). Samples were then centrifuged for 10 min (4°C) at 8,000 × g, snap-frozen with liquid nitrogen, and sent to Sangon Biological Co., Ltd., for RNA-Seq analysis. Briefly, total RNA was extracted from these yeast, mRNA was enriched from these extracts, and the purified mRNA was fragmented and utilized as a template for cDNA synthesis. After preparation, cDNA libraries were sequenced with an Illumina HiSeq device (Illumina, CA, United States).

### Functional annotation of DEGs

Base calling was used to turn the raw image data from Illumina sequencing into sequence data. FastQC (version 0.11.2) was used to evaluate the quality of sequenced data. Raw reads were filtered using Trimmomatic (version 0.36) through the several steps: (1) The removal of sequences with N bases; (2) The removal of adaptor sequences when present in detected reads; (3) The removal of low-quality bases from reads in a 3′ to 5′ direction (Q < 20); (4) The removal of low-quality bases from reads in a 5′ to 3′ direction (Q < 20); (5) The use of a sliding window method to remove base values < 20 from read tails (window size: 5 bp); (6) Removing reads with reads length less than 35 nt and paired reads. The remaining clean data were used for further analysis.

HISAT2 (version 2.1.0) was used to align the clean reads with the reference genome. Blast2GO (Götz et al., 2008) was used to annotate gene functions in the Gene Ontology (GO) database (Harris et al., 2004), and the euKaryotic Orthologous Groups (KOG) database was used to phylogenetically classify the encoded proteins, and the associated metabolic pathways were annotated using the Kyoto Encyclopedia of Genes and Genomes (KEGG) database (Kanehisa and Goto, 2000).

StringTie (version 1.3.3b) was used to assemble transcripts and GffCompare (version 0.10.1) was used to align these with known gene modules to detect novel transcript regions. To identified significant DEGs, the following settings were used: *q*-value < 0.05, Log_2_| FoldChange| > 1 (Reiner et al., 2003; Trapnell et al., 2011, 2013).

### qRT-PCR

An qRT-PCR approach was employed to confirm the differential expression of key genes of interest identified by RNA-Seq in the Wyslmt strain. These qRT-PCR assays were performed using a 4S Red Plus Nucleic Acid Stain (A606695, Sangon Biotech, Shanghai, China) with the primers being listed in Table 1. Briefly, control Wyslmt cells and cells cultured in the presence of Diquat (2,500 mg/L) were grown for 24 h at 28°C and 180 rpm. Total RNA was extracted from these cells using the same protocols employed prior to RNA-seq analyses. Random primers and Maxima Reverse Transcriptase (EP0743, Thermo Scientific, Shanghai, China) were then used to prepare cDNA, which was used as a template for qRT-PCR. Three biological replicates were analyzed per sample, and the 2^–ΔΔ*CT*^ method was used to assess relative gene expression (Cheng et al., 2018).

**TABLE 1:** Primer sequences in the present study.

Gene	Primer sequences (5′–3′) (Forward/Reverse)	Product length (bp)
GAPDH	TCTTCGGACGCTCCTATGTT GGTCATCAAACCCTCCTCAA	156
DN671_c0_g12	CGGTAGTCGGAACTTCTGTATTG AGTGTTGTAATCGTGTTCATAGAGC	101
DN678_c2_g13	CTAAAGTGGCTCCAACTGTGAAC AAGGAGACATTATCATTACCGAAAC	132
DN650_c0_g4	GCACGCCAACAACGAAGA GAGAGTTTTACACTTCACAATGGGT	169
DN678_c2_g10	ATGTTGCCTCGTATGGATGC CATATCTCATCAGTTTTCAGCATTT	87
DN95_c0_g1	GTCACCTCAGTATGCAGATTCTTG CTGATGGCTGCCCTGCTT	204
DN1001_c0_g1	TCGGCATCGGCATTAGCA GGAACTGGAACCAAGACGAGAG	149
DN640_c0_g18	CGTAGTCAGGTTGGTCCGTAA AATGGTCTCGTTGATTCCGTAGT	114
DN573_c0_g4	ATCTATGCCAATGTGCCAATAACTC ACAGCAGCGAATGGAAGGTA	163
DN554_c0_g13	CGATACACGCACCGAAGATAGATA CGCAACATTCAAGAACTACCTCAG	167
DN676_c0_g3	GCCGAGCCAAGCGATACT TCCAGCAAGAACACCACAGAA	186

### Degradation candidate gene cloning and expression

Following the cloning and expression testing of the 10 candidate genes indicated above, the gene *DN676 c0 g3*—also known as *DN676*—was chosen for further study. The *DN676* gene was combined with the expression vector pET-4T-1 and digested with *Bam*HI and *Sma*I to create the recombinant plasmid pET-*DN676*. This plasmid was then transformed into capable *E. coli* BL21 (DE3) cells *via* heat shock (37°C, 200 rpm). Isopropy-D-thiogalactoside (IPTG) was added at a final concentration of 0.5 mM at 20 and 37°C when the OD_600_ value reached 0.6. Cells were collected and centrifuged at 4,000 rpm for 10 min after being cultured for 2 h. The protein was then added to the buffer for SDS-polyacrylamide gel electrophoresis (SDS-PAGE) separation after the supernatants had been removed.

### Functional verification of the ability of the *DN676* gene to degrade Diquat

To confirm the relationship between this gene and Diquat degradation, the BL21-pET strain served as a control group, while the BL21-pET-*DN676* group was the treatment group. These BL21-pET and BL21-pET-*DN676* bacteria were cultured until reaching the logarithmic phase of growth (OD_600_ = 0.6), at which point a 1% inoculum was transferred into 100 mL of DLB containing Diquat (100 mg/L). The concentration of Diquat and bacterial growth were measured on days 1, 3, 5, and 7 of incubation at 37°C with constant agitation (200 rpm). Bacterial growth was monitored by measuring the absorbance of the culture supernatant at 600 nm using a TU-1901 spectrophotometer (Beijing Purkinje General Instrument Co., Ltd., China). The concentration of Diquat was measured *via* HPLC. Experiments were repeated in triplicate.

### Statistical analysis

DEGseq (version 1.26.0) was used to detect DEGs (*q*-value < 0.05, Log_2_| FoldChange| > 1) in RNA-seq studies between the control group and the treatment group. ANOVA (SPSS version 21.0) were used to analyze KEGG enrichment data, with *P* < 0.05 being considered the threshold of significant pathway enrichment.

## Results

### Isolation and identification of the Diquat-degrading Wyslmt yeast strain

**FIGURE 1:**
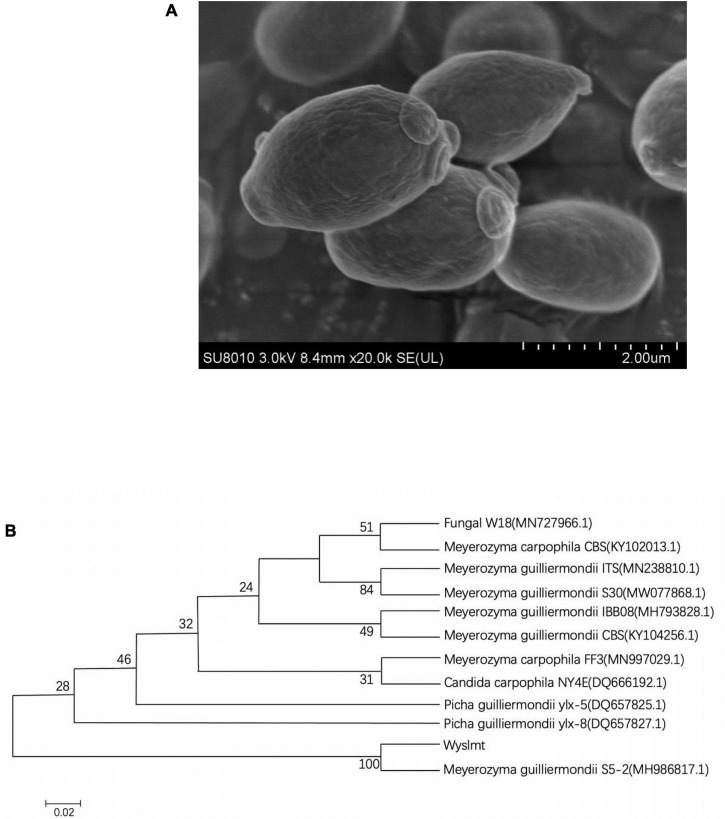
Scanning electron micrograph of the Wyslmt strain **(A)**. Phylogenetic tree based on the 26S rDNA gene sequences from the Wyslmt yeast strain **(B)**. The neighbor-joining method was used to construct a phylogenetic tree (bootstrap number = 1,000), with the scale bar denoting 0.02 substitutions per nucleotide position.

The Wyslmt yeast strain, which exhibited robust Diquat degrading activity, was isolated and selected for further investigation. Physiological and morphological characterization indicated that Wyslmt cells were ovoid and without flagella, consistent with their characterization as a yeast strain (Figure 1A).

The 26S rDNA gene isolated from Wyslmt yeast was 579 bp in length, and a comparison of this sequence to other sequences available in the GenBank database indicated it to be 100% homologous to sequences form *Meyerozyma guilliermondii* S5-2. A phylogenetic tree was then constructed *via* the neighbor-joining approach. In the resultant tree (Figure 1B), this Wyslmt strain clustered with *Meyerozyma guilliermondii*.

### Biodegradation characteristics of the Wyslmt yeast strain

**FIGURE 2:**
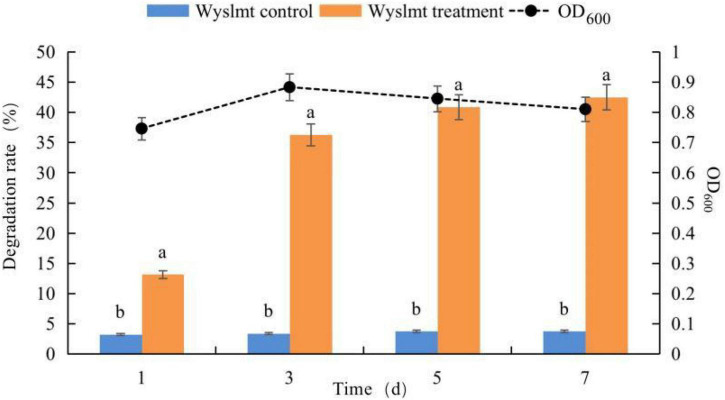
Growth of the Wyslmt strain and associated Diquat degradation. Error bars represent the standard deviation for three replicate samples. Different letters indicate significant differences between treatments (*P* < 0.05).

Next, we assessed the ability of this Wyslmt strain to degrade Diquat. Samples collected from the culture medium were analyzed for growth at 600 nm *via* ultraviolet spectrophotometry and also subjected to HPLC analyses. These yeast cells grew in accordance with a typical growth curve, and the concentration of Diquat in the culture medium decreased throughout the experimental period (Figure 2). In total, 42.51% of the provided Diquat (100 mg/L) was removed by this strain after a 7-day incubation period.

### RNA-seq identification of genes differentially expressed in Wyslmt in the context of Diquat degradation

In total, 44,132,249 and 47,354,547 raw RNA-Seq reads were obtained from the Diquat-treated and control groups, respectively. After filtering, 42,760,279 and 45,769,503 clean reads of 5,859,601,868 bp (GC content: 46.27%) and 6,320,620,062 bp (GC content: 46.19%) were obtained for the treatment and control groups, respectively. The Q20 and Q30% values for the clean data were 98.53 and 98.48% for the treatment group, respectively, and 96.20 and 96.28% for the control group, respectively. Following the removal of sequences < 20 bp in length, 2,931 non-reductant transcripts were retained. These differentially expressed genes were next subjected to GO and KEGG analyses aimed at elucidating their roles in the context of Diquat biodegradation.

### Functional annotation and metabolic pathway classification in the Wyslmt strain

The GO database was used to annotate 2,310 genes of the Wyslmt strain in total (Supplementary Figure 1). In the GO database, a significant number of genes were annotated in 8 out of the 64 groups of GO terms divided in the 3 categories (molecular functions, biological processes, and cellular components) of the gene products, including the binding (1,361 genes assigned, accounting for 58.92% of the 2,310 genes annotated), catalytic activity (1,301, 56.32%), cellular process (1,743, 75.45%), metabolic process (1,527, 66.10%), cell (1,997, 86.45%), cell part (1,995, 86.36%), organelle (1,652, 71.52%), and organelle part (1,124, 48.66%) terms. In contrast, relatively few genes (1–2) were associated with 9 groups of GO terms including the chemoattractant activity, protein tag, channel regulator activity, receptor regulator activity, behavior, biological phase, cell aggregation, cell killing, and extracellular matrix terms.

Using sequence homology as the basis, 1,784 genes in all were mapped onto 24 potential protein categories in the KOG database (Supplementary Figure 2). The greatest numbers of mapped genes were connected with eight gene function classes, including the RNA processing and modification (100 genes assigned, accounting for 5.61% of the 1,784 mapped genes), energy production and conversion (130, 7.29%), amino acid transport and metabolism (134, 7.51%), carbohydrate transport and metabolism (108, 6.05%), translation, ribosomal structure and biogenesis (191, 10.71%), general function prediction only (211, 11.83%), signal transduction mechanisms (105, 5.89%), and intracellular trafficking, secretion, and vesicular transport (113, 6.33%) classes. In contrast, just 0–1 mapped genes were associated with the cell motility class.

In the KEGG database, a total of 895 genes were categorized into 32 metabolic pathways to identify their biological roles (Supplementary Figure 3), with the translation pathway being associated with the greatest number of these genes (154, accounting for 17.21% of the 895 classified genes), followed by 127 genes (14.19%) associated with signal transduction and 124 genes (13.85%) associated with carbohydrate metabolism. Many other metabolic pathways were associated with fewer than 10 genes, including the membrane transport (8), development (9), environmental adaptation (9), and sensory system (5) pathways.

### Identification and analysis of differentially expressed genes in the Wyslmt strain

**FIGURE 3:**
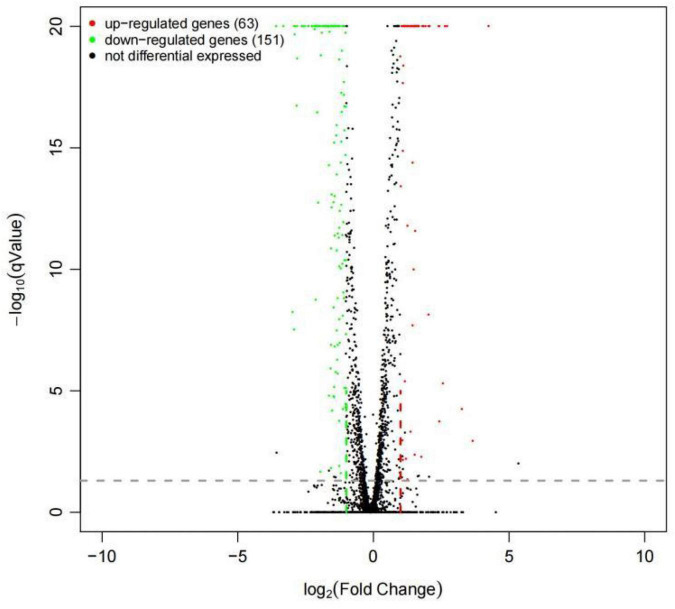
Differential gene expression in Wyslmt cells in the context of Diquat degradation. Individual points correspond to specific genes, with upregulated, downregulated, and non-differentially expressed genes being shown in red, green, and black, respectively.

To have a comprehensive understanding of the Wyslmt strain’s transcriptome response to the degradation of Diquat, DEGseq (version 1.26.0) was used to further analyze differences in gene expression profiles between the treatment and control groups. In total, 214 DEGs (*q*-value < 0.05, Log_2_| FoldChange| > 1) were identified between the treatment and control groups, of which 63 (29.44%) were upregulated and 151 (70.56%) were downregulated. The upregulated genes may play a positive role in the metabolic processing of Diquat, while the downregulated genes may be associated with the toxicity of Diquat to this Wyslmt strain (Figure 3).

**FIGURE 4:**
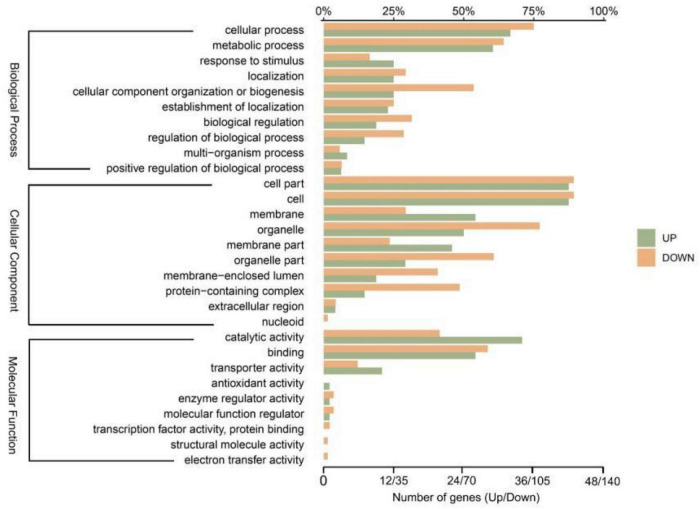
GO annotation histograms for the Wyslmt strain’s DEGs. The precise classification of GO terms is represented by the vertical coordinates. The three brackets show three secondary GO database classifications (biological process, cellular component, and molecular function). The percentage of genes annotated with GO terms in relation to all genes annotated with GO terms is shown by the upper abscissa. The number of genes with GO annotations is shown in the bottom abscissa. Horizontal green and yellow bars, respectively, show the up-regulated and down-regulated genes.

By mapping the genes onto corresponding GO terms to explore the 214 DEGs’ functions (Figure 4). In total, the mapping of 185 DEGs onto 64 groups of GO terms in three main categories (molecular functions, cell components, and biological processes). Genes differentially expressed in response to Diquat were associated with regulatory and metabolic processes, including the cellular process, metabolic process, cell part, cell, organelle, binding, and catalytic activity GO terms. Most DEGs were associated with the biological process terms metabolic process and cellular process. Specifically, 29 upregulated genes and 90 downregulated genes were related to the metabolic process GO term, while 32 upregulated genes and 105 downregulated genes were associated with the cellular process term. These results demonstrated that the Wyslmt strain’s metabolic functions were impacted by growth in Diquat-containing medium, with the DEGs associated with these GO terms potentially being involved in the process of Diquat degradation. Furthermore, the catalytic activity GO term was associated with 34 upregulated and 58 downregulated genes, while the binding term was associated with 26 upregulated and 82 downregulated genes. These results showed a possible connection between the DEGs linked to these two GO terms and the Diquat-associated catalytic and binding activity in the Wyslmt strain.

**FIGURE 5:**
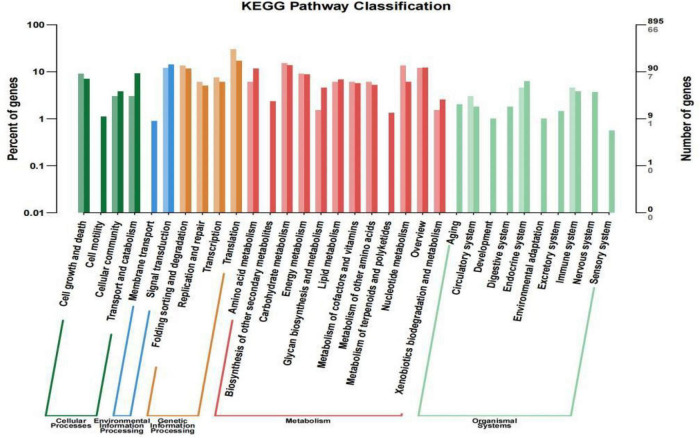
KEGG pathway enrichment analysis results for the Wyslmt strain. Functional categories are shown on the horizontal axis, while the vertical axis corresponds to the number of genes per category (right) and the percentage of the total number of annotated genes (left). Different categories are marked with different colors. Differentially expressed genes and all genes are shown in lighter and darker colors, respectively.

These 214 DEGs were also subjected to a KEGG enrichment analysis aimed at identifying associated metabolic pathways (Figure 5). The results of these analyses revealed that the 32 enriched metabolic pathways (*P*-value < 0.05) between the treatment and control groups were associated with five categories, including Environmental Information Processing (EIP), Genetic Information Processing (GIP), Cellular Processes (CP), Organismal Systems (OS), and Metabolism (M) (Figure 5). A total of 66 DEGs were associated with 32 KEGG pathways, and most of these DEGs were enriched in the metabolism category, including amino acid metabolism, carbohydrate metabolism, energy metabolism, lipid metabolism, metabolism of cofactors and vitamins, metabolism of other amino acids, and nucleotide metabolism.

Of these DEGs, 63 upregulated genes may be involved in the process of Diquat degradation. According to annotation results, numerous genes are probably predicted to encode transfer/transport proteins or enzymes that might either directly or indirectly contribute to the metabolism of Diquat before being involved in its degradation. Based on Log_2_| FoldChange| values for these of these 63 upregulated DEGs, we screened 10 candidate genes that may facilitate Diquat degradation (Table 2). Among these, gene *DN573_c0_g4* was identified as allantoicase and associated with purine metabolism, gene *DN554_c0_g13* was identified as Choline/ethanolaminephosphotransferase 1 and associated with phosphonate and phosphinate metabolism, glycerophospholipid metabolism, and ether lipid metabolism, and gene *DN95_c0_g1* was identified as gamma-glutamyltransferase and associated with cyanoamino acid metabolism, glutathione metabolism, and taurine and hypotaurine metabolism. While the 7 remaining genes were not specifically associated with any metabolic pathways, we cannot rule out the potential that these enzymes are involved in the degradation of Diquat.

**TABLE 2:** DEGs related to Diquat biodegradation by the Wyslmt strain.

Gene	Annotation	GO	KEGG
**Upregulated**			
DN640_c0_g18	Amino acid transporters	Cellular component; membrane part	−
DN573_c0_g4	Allantoicase	Biological process; metabolic process	Purine metabolism
DN671_c0_g12	L-type amino acid transporter.	Biological process; biological regulation	−
DN678_c2_g13	Thiamine pyrophosphate-requiring enzyme	Molecular function; binding	−
DN554_c0_g13	Choline/ethanolaminephosphotransferase 1	Cellular component; organelle part	Phosphonate and phosphinate metabolism; Glycerophospholipid metabolism; Ether lipid metabolism
DN650_c0_g4	NADH-dependent fumarate reductase	Molecular function; binding	−
DN1001_c0_g1	Drug resistance transporter, Bcr/CflA subfamily	Biological process; biological regulation	−
DN678_c2_g10	Pyruvate decarboxylase and related thiamine pyrophosphate-requiring enzymes	Molecular function; binding	−
DN676_c0_g3	PDR transporter	Molecular function; transporter activity	−
DN95_c0_g1	Gamma-glutamyltransferase	Biological process; metabolic process	Cyanoamino acid metabolism; Glutathione metabolism; Taurine and hypotaurine metabolism

The “−” symbol indicates the absence of annotated functions.

### qRT-PCR validation

**FIGURE 6:**
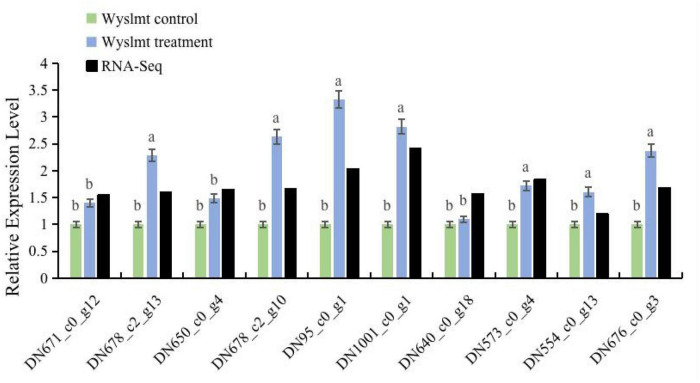
The relative mRNA expression levels of DEGs in both the treatment and control groups. Black bars represent log_2_| FoldChange| (treatment group/control group) values from RNA-seq analyses. Error bars represent the standard deviation for three replicate samples. Different letters indicate significant differences between treatments (*P* < 0.05).

Next, the mRNA expression levels of the Diquat degrading genes were measured to validate the transcriptome results *via* qRT-PCR, as shown in Figure 6. To determine the expression levels of these genes during the biodegradation process, the RNA-Seq results were utilized as a reference. The resultant | Log_2_FC| values (>1) confirmed the upregulation of the genes in the treatment group identified *via* RNA-Seq. The Diquat concentration utilized for preparing samples for RNA-Seq and qRT-PCR was 2,500 mg/L, and RNA extraction was performed following a 24 h culture period to further validate RNA-Seq findings. As the Wyslmt strain was capable of degrading Diquat when cultured for 24 h, the genes upregulated during this process probably connected to the Wyslmt strain’s ability to metabolize Diquat or to increase its tolerance to Diquat. Overall, the mRNA expression levels for the seven genes under investigation are considerably greater in the treatment group than in the control group (*DN678_c2_g13*, *DN678_c2_g10*, *DN95_c0_g1*, *DN1001_c0_g1*, *DN573_c0_g4*, *DN554_c0_g13*, and *DN676_c0_g3*). Accordingly, we hypothesize that these seven genes may be crucial in the Wyslmt strain’s ability to degrade Diquat, in line with our above findings.

### Cloning and prokaryotic expression of the *DN676* gene

**FIGURE 7:**
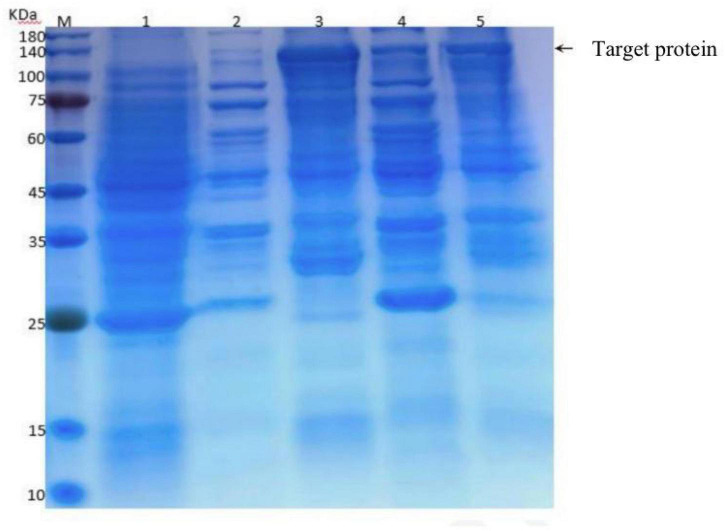
Induced expression of the recombinant plasmid at different temperatures. M: Protein marker; 1: Total protein before induction; 2–5: BL21-pET-DN676-induced supernatants at 20°C, precipitates at 20°C, supernatants at 37°C, and precipitates at 37°C.

Other qualified candidates were cloned and found to not increase Diquat degradation. The functions of this gene were predicted using the https://www.ncbi.nlm.nih.gov/Structure/cdd/wrpsb.cgi website, which identified *DN676* as a member of the Pleiotropic Drug Resistance (PDR) family. A clear band was evident at 140 kDa, with its molecular weight being close to the predicted molecular weight (GST fusion protein 26 kDa + 112 kDa = 138 kDa), confirming the successful expression of the this protein in *E. coli* BL21, although it was primarily present in the precipitate in the form of inclusion bodies (Figure 7).

### Identification of *DN676* as a gene associated with Diquat degradation

**FIGURE 8:**
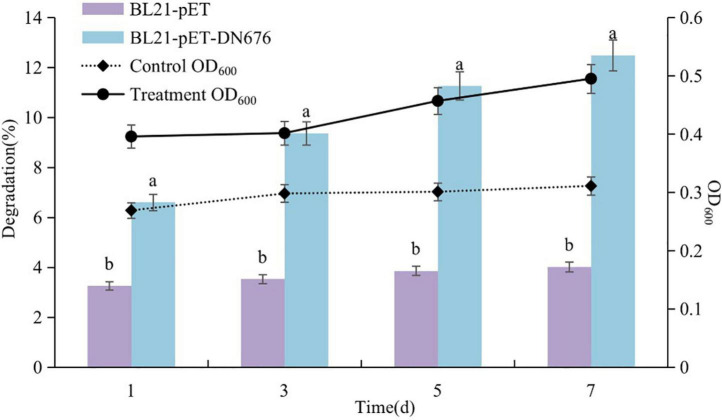
Growth and Diquat degradation of BL21-pET and BL21-pET-*DN676* strains. Error bars represent the standard deviation of three replicates. Different letters indicate significant differences between different treatments (*P* < 0.05).

Samples were collected from culture medium and growth was assessed at 600 nm *via* ultraviolet spectrophotometry, while Diquat levels were measured *via* HPLC (Figure 8). A typical growth curve was observed for the BL21-pET-*DN676* strain, whereas the BL21-pET microbes exhibited negligible growth under these conditions. The concentration of Diquat decreased throughout the experimental period such that 12.49% of Diquat (100 mg/L) was removed by the BL21-pET-*DN676* strain and 4.01% was removed by the BL21-pET strain after a 7-day incubation period. These results thus confirmed the Diquat-degrading activity of the *DN676* gene.

## Discussion

Funderburk (1969) has previously described a strain of *Lipomyces starkeyi* capable of degrading Diquat, but their experimental details remain to be published. Current evidence suggests that *L. starkeyi* can degrade both paraquat and Diquat, utilizing them as a sole nitrogen source to support cell growth. In the present study, a novel yeast strain was isolated from soil exposed to long-term Diquat treatment. In total, 42.51% of Diquat (100 mg/L) was removed by this strain after a 7-day incubation, with this strain thus exhibiting a high degree of Diquat degradation efficiency. Current research on Diquat degradation has focused on the isolation and the characterization of specific microorganisms. In contrast, there have been few studies of the metabolic genes responsible for Diquat degradation. A greater knowledge of the factors that regulate gene expression in specific species may be gained by analysis of microbial RNA-Seq data that reveals the mechanisms of action for particular regulatory genes. As such, RNA-Seq was performed in this study to assess changes in gene expression in this yeast strain over the course of Diquat degradation, revealing 63 and 151 upregulated and downregulated genes, respectively. KEGG pathway enrichment analysis revealed these genes to be most highly enriched in the carbohydrate metabolism pathway. Functional annotation, gene expression analyses, and qRT-PCR assays further revealed *DN676* as a candidate Diquat-degrading gene.

RNA-Seq is an efficient means of mining for degraded genes. For example, Zhang et al. (2019) employed an RNA-Seq approach to explore the mechanisms whereby Klebsiella jilinsis 2N3 was able to degrade chlorimuron-ethyl, finding it to do so through a mechanism linked to the sulfur metabolism pathway, with genes encoding carboxylesterases, monooxygenases, glycosyltransferases, and cytochrome P450 being significantly upregulated in treated cells in the context of chlorimuron-ethyl degradation. Through subsequent knockout experiments, they determined that cytochrome P450 enzymes and the *Kj-CysJ* and *Kj-SsuD* genes are likely to play central roles in chlorimuron-ethyl degradation (Zhang et al., 2019). Herein, a series of RNA-Seq, qRT-PCR, GO, and KEGG enrichment analyses led to the identification of candidate genes associated with Diquat degradation. These analyses revealed that the carbohydrate metabolism pathway was the most profoundly enriched in the context of Diquat treatment, in line with the similar findings pertaining to sulfur metabolism in the study conducted by Zhang et al. (2019). The *DN676* gene was then screened as a candidate associated with Diquat degradation. In contrast to the cytochrome P450 genes identified by Zhang et al. (2019), *DN676* is a member of the PDR family, and its function is poorly characterized.

With the exception of Adp1p, PDR members are all full-size ABC proteins. The PDR family is unusual in that it is only found in fungi and plants. Herbicides, phospholipids, peptides, steroids, and anticancer medications are all transported *via* specific PDR-transporters (Schüller et al., 2003). Yeast cells can quickly counteract toxic environmental challenges through efficient detoxification systems such as the PDR machinery. It’s critical to understand that PDR not only makes cells hypertolerant to numerous unrelated exogenous medications or xenobiotics, but also shields cells against the negative effects of harmful endogenous metabolites (Helmut and Karl, 2006). Several *S. cerevisiae* PDR genes, such as PDR5 and SNQ2, have been functionally characterized in detail. They confer resistance to a large number of toxic compounds with no, or few common structural or functional properties, such as fungicides, herbicides, pesticides, antibiotics, and detergents. The cytotoxic and mutagenic properties of several of these compounds suggest the involvement of these transporters in cell detoxification and cell resistance (Bauer et al., 1999; Rogers et al., 2001). PDR transporters are also involved in the fungicide resistance of pathogenic fungi such as *Candida albicans* (Prasad and Kapoor, 2005) or *Penicillium digitatum* (Nakaune et al., 1998). In the present study, the outcomes demonstrated that the growth of the BL21-pET and BL21-pET-*DN676* strains steadily grew over time, and there was a substantial difference between the two (*P* < 0.05). This indicated that the *DN676* gene had increased the resistance to Diquat and had the same result as above. The molecular processes through which members of the ABC protein family members function have become better understood as a result of intensive research efforts in recent years. Many eukaryotic ABC pump proteins’ cellular substrates and physiological roles, however, are yet unknown. Given as PDR transporters remove a large number of potentially harmful metabolites and hundreds of structurally and functionally unrelated cytotoxic chemicals, detoxification is still one of their most plausible physiological activities (Kolaczkowska and Goffeau, 1999). Few studies have explored whether these proteins degrade herbicides or change their structures in the context of detoxification, leading to the selection of *DN676* as a candidate Diquat degradation gene for functional verification. In these analyses, when the pET-*DN676* vector was expressed in *E. coli* BL21, this strain was able to remove 12.49% of provided Diquat (100 mg/L) over the course of a 7-day incubation. The *DN676* gene increased the degradation of Diquat by BL21, thus confirming that the *DN676* gene is related to the degradation of Diquat. However, how the *DN676* gene facilitates Diquat degradation warrants further study. Even so, these results suggest that PDR transporters may play an important role in herbicide degradation, potentially opening an entirely new field of research with promising implications for the fields of biotechnology and agriculture.

Zhang et al. (2019) verified the relationship between their genes of interest and chlorimuron-ethyl degradation *via* a genetic knockout’ approach, we instead verified the role of our target gene of interest by expressing it in *E. coli* and generated an engineered strain with the ability to degrade Diquat. This method is simple, and allowed for the rapid production strains with specific functions. *E. coli* strains are very common, and their application in agricultural contexts will not adversely impact local flora. As such, these engineered microbes may represent a valuable tool for future biodegradation and bioremediation efforts. In addition, further RNA-Seq analyses of Diquat degrading strains may provide new insights into the environmental effects of Diquat stress and may facilitate the discovery of new functional genes of interest.

## Conclusion

In summary, the *Meyerozyma guilliermondii* Wyslmt yeast strain was herein isolated from soil that had been exposed to Diquat for an extended period. In total, 42.51% of Diquat (100 mg/L) was removed by this strain after a 7-day incubation. The molecular mechanisms whereby these yeast degrade Diquat were then explored *via* RNA-Seq. KEGG enrichment analyses indicated that the carbohydrate metabolism pathway exhibited the highest enrichment ratio, and qRT-PCR demonstrated that the treatment group’s levels of mRNA expression of the seven genes during the Diquat degradation process were significantly greater than those of the control group. *E. coli* gene transfer experiments further demonstrated that when a pET-*DN676* vector was expressed in *E. coli* BL21, 12.49% of Diquat (100 mg/L) was removed by this strain after a 7-day incubation. As such, these results may highlight an effective approach to generating engineered microbial strains with the ability to degrade Diquat.

## Data availability statement

The datasets presented in this study can be found in online repositories. The names of the repository/repositories and accession number(s) can be found in the article/Supplementary material.

## Author contributions

FW and BT: conceptualization and methodology. FW: validation, data curation, and writing—original draft preparation. FW, YH, LK, JG, XS, and BT: writing—review and editing. All authors have read and agreed to the published version of the manuscript.
