# Supplementary material for: RNA-sequencing analysis of the Diquat-degrading yeast strain Meyerozyma guilliermondii Wyslmt and the discovery of Diquat degrading genes
Source: Front Microbiol. 2022 Sep 2;13:993721. doi: 10.3389/fmicb.2022.993721 (PMC9478375; doi:10.3389/fmicb.2022.993721)
Supplement: Supplementary file 1 [file Data_Sheet_1.docx]

Supplementary Material


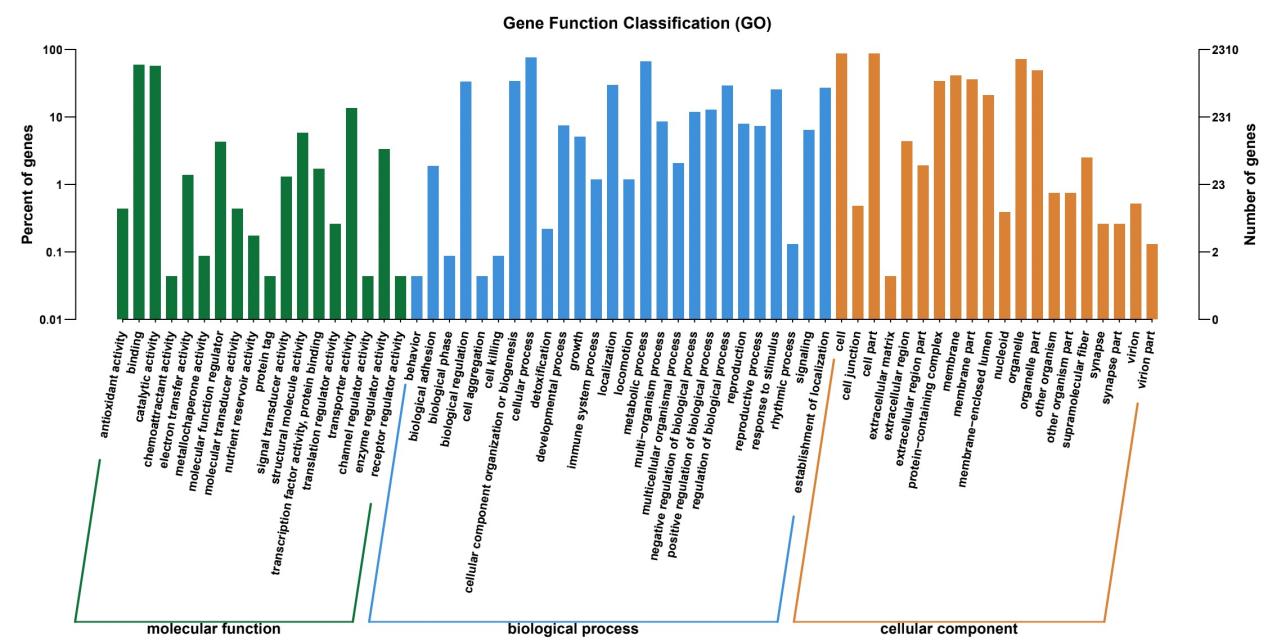


**Figure S1.** GO annotation distributions for Diquat degradation-related genes. The horizontal axis corresponds to the secondary GO classification, while the vertical axis denotes the number of genes in that classification (right) and its percentage relative to the total number of annotated genes (left). Different colors represent different orthologs.


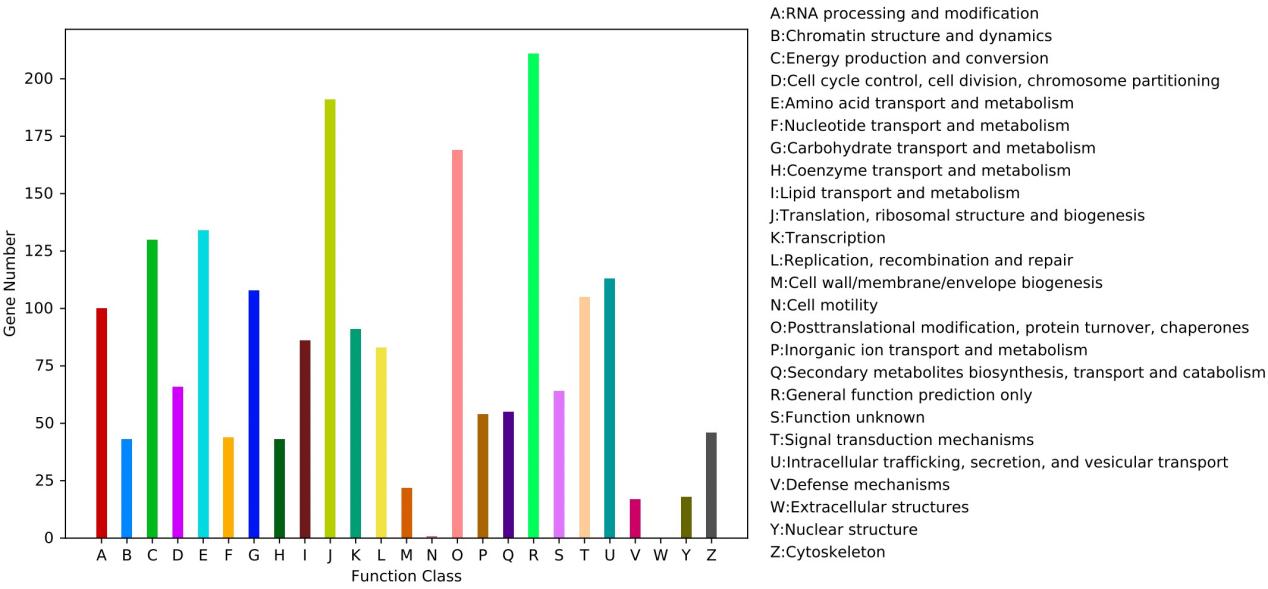


**Figure S2.** KOG classification histogram. Each color on the horizontal axis represents the functional classification of a KOG, as annotated in the legend on the right. The vertical axis corresponds to the number of genes annotated to this category.


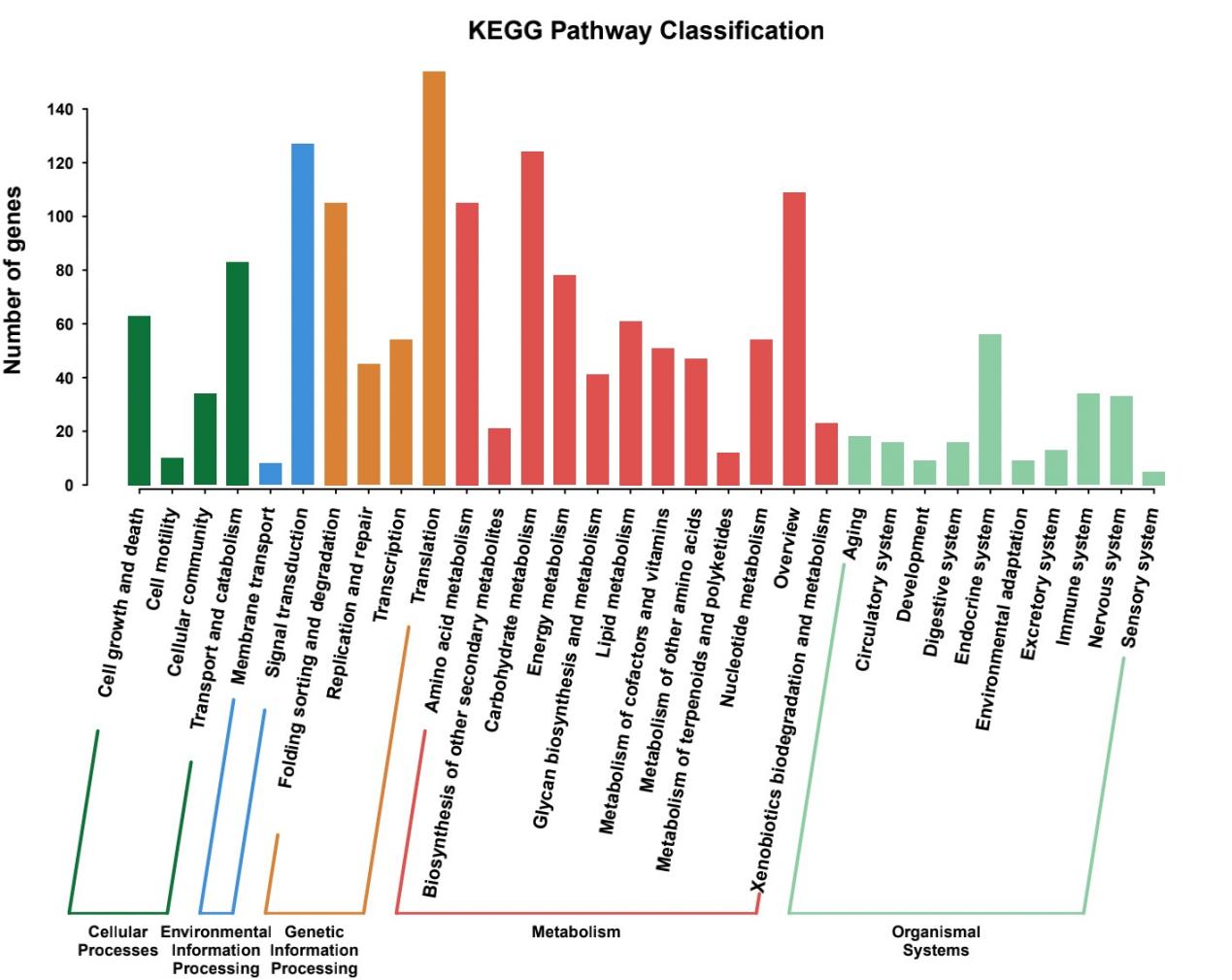


**Figure S3.** KEGG Pathway classification histogram. The horizontal axis denotes the specific KEGG pathway, while the vertical axis indicates the number of genes annotated to the pathway.
